# Integrative analysis of clinical and bioinformatics databases to identify anticancer properties of digoxin

**DOI:** 10.1038/s41598-019-53392-y

**Published:** 2019-11-12

**Authors:** Satoshi Yokoyama, Yasuhiro Sugimoto, Chihiro Nakagawa, Kouichi Hosomi, Mitsutaka Takada

**Affiliations:** Division of Clinical Drug Informatics, School of Pharmacy, Kindai University, 3-4-1 Kowakae, Higashiosaka City, Osaka 577-8502 Japan

**Keywords:** Databases, Literature mining, Microarrays, Phenotypic screening

## Abstract

Cardiac glycosides, such as digoxin, inhibit Na^+^/K^+^-ATPases and cause secondary activation of Na^+^/Ca^2+^ exchangers. Preclinical investigations have suggested that digoxin may have anticancer properties. In order to clarify the functional mechanisms of digoxin in cancer, we performed an integrative analysis of clinical and bioinformatics databases. The US Food and Drug Administration Adverse Event Reporting System and the Japan Medical Data Center claims database were used as clinical databases to evaluate reporting odds ratios and adjusted sequence ratios, respectively. The BaseSpace Correlation Engine and Connectivity Map bioinformatics databases were used to investigate molecular pathways related to digoxin anticancer mechanisms. Clinical database analyses suggested an inverse association between digoxin and four cancers: gastric, colon, prostate and haematological malignancy. The bioinformatics database analysis suggested digoxin may exert an anticancer effect via peroxisome proliferator-activated receptor α and apoptotic caspase cascade pathways. Our integrative analysis revealed the possibility of digoxin as a drug repositioning candidate for cancers.

## Introduction

Cardiac glycosides have been traditionally used for treatment of heart failure and cardiac arrhythmias. The mechanisms of cardiac glycoside action involve inhibition of Na^+^/K^+^-ATPases and secondary activation of Na^+^/Ca^2+^ membrane exchangers. Although some preclinical investigations have suggested that cardiac glycosides may have anticancer properties^1–5^, others have reported they exacerbate cancer risk in clinical settings^6^. The basic structure of cardiac glycosides is similar to that of estradiol. One cardiac glycoside, digoxin, has the ability to bind oestrogen receptors and increases the risk of oestrogen-sensitive breast and uterine cancers^7–10^. Thus, the effect of cardiac glycosides in cancer remains controversial.

Recently, drug repositioning approach has been remarkably employed as conventional drug development requires high cost and long duration. Various methods utilising big data have been developed to identify unexpected associations between existing drugs and the outcomes of interests^11–15^. Big data contains real-world data such as the US Food and Drug Administration Adverse Event Reporting System (FAERS) and claims database or bioinformatics database such as Omics data and are widely available to researchers. Disproportionality analysis (DPA) and sequence symmetry analysis (SSA) using real-world data have been used in pharmacovigilance. Integrated analysis using DPA and SSA can enhance signal detection because SSA detects any additional true-positive signals that are not detected using DPA alone^16^. These analytical methods can detect a risk signal, indicating a possible causal relationship between drug exposure and an adverse event. Conversely, inverse signals detected using DPA and SSA generally have been thought to have no implication. However, we previously noted that inverse signals between a target drug and an adverse drug reaction suggest potential alternative therapeutic opportunities^17^, and several reports have evaluated these inverse associations for drug repositioning approaches^18,19^. Recently, drug repositioning studies using clinical real-world data have been conducted^20,21^. Moreover, bioinformatics databases are publicly available and are considered as a valuable source of data regarding phenotypes. Specifically, bioinformatics databases containing microarray gene expression profiles have been used for seeking novel molecular mechanisms^18,22^. In turn, the value of an integrative approach using both real-world data and bioinformatics databases was recently reported^23^. In the present study, functional relationships between digoxin and cancer were investigated by integrative analysis of multiple, large clinical and bioinformatics databases.

## Results

### Association between digoxin and cancer based on FAERS and Japan Medical Data Center (JMDC) claims databases

A total of 300,541 drug-reaction pairs for digoxin were found in the FAERS database. The association between digoxin use and cancers based on FAERS are shown in Table 1. In the analyses of individual cancers, significant inverse signals in both reporting odds ratios (RORs) and information components (ICs) were found for digoxin with gastric, colorectal, pancreatic, breast, ovarian, prostate and bladder cancers as well as melanoma and haematological malignancy. There were no positive signals in this DPA using the FAERS database.

**Table 1:** The association between digoxin use and cancers based on FAERS.

Cancer	Cases	Non-cases	ROR	95%CI		IC	95%CI	
				Lower	Upper		Lower	Upper
Esophageal cancer	18	8,077	0.73	0.46	1.16	−0.43	−1.08	0.22
Gastric cancer	26	14,758	0.58*	0.39	0.85	−0.76*	−1.31	−0.22
Colorectal cancer	88	48,122	0.60*	0.49	0.74	−0.73*	−1.03	−0.43
Pancreatic cancer	83	38,913	0.70*	0.57	0.87	−0.51*	−0.81	−0.20
Lung cancer	79	28,028	0.93	0.74	1.16	−0.11	−0.42	0.21
Melanoma	30	24,082	0.41*	0.29	0.59	−1.26*	−1.77	−0.75
Breast cancer	197	203,451	0.37*	0.32	0.42	−1.44*	−1.64	−1.24
Uterine cancer	38	18,771	0.77	0.56	1.05	−0.38	−0.83	0.08
Ovarian cancer	22	16,716	0.50*	0.33	0.76	−0.97*	−1.56	−0.38
Prostate cancer	101	41,237	0.64*	0.53	0.78	−0.63*	−0.91	−0.35
Bladder cancer	57	40,052	0.47*	0.36	0.61	−1.08*	−1.45	−0.71
Hematological malignancies	429	229,446	0.61*	0.56	0.68	−0.70*	−0.84	−0.56

Cases: number of reports in digoxin, Non-cases: all reports of adverse drug reactions other than digoxin.

FAERS, FDA’s Adverse Event Reporting System; IC, information component; CI, confidence interval; ROR, reporting odds ratio.

*: significant inverse signal.

The characteristics of the study population in the JMDC claims database are summarised in Table S1. The number of claims pertaining to digoxin during the study period was 52,828. Among 3,035 digoxin users, 1,297 incident users who received their first digoxin prescription were identified. Table 2 shows the associations between digoxin use and cancers. Digoxin use was inversely associated with diagnoses of oesophageal, gastric, colorectal, lung and prostate cancers as well as haematological malignancy. Analyses of pancreatic, breast, uterine, ovarian and bladder cancer diagnoses showed no significant signal associations with digoxin use. Consequently, analyses of both FAERS and JMDC claims databases revealed significant inverse signals in gastric cancer, colorectal cancer, prostate cancer and haematological malignancy related to digoxin use.

**Table 2:** Event sequence symmetry analysis: the associations between digoxin and cancers.

Cancer	Incident users	Cases with cancer	Interval	Diagnosis of cancer		ASR	95%CI	
			(months)	Last	First		Lower	Upper
Esophageal cancer	4,782	16	12	1	11	0.08*	0.00	0.57
			24	2	12	0.14*	0.02	0.62
			36	2	14	0.11*	0.01	0.48
			48	2	14	0.10*	0.01	0.45
Gastric cancer	57,140	159	12	27	45	0.56*	0.33	0.92
			24	43	63	0.60*	0.40	0.90
			36	53	74	0.60*	0.41	0.86
			48	55	78	0.56*	0.39	0.80
Colorectal cancer	69,173	217	12	45	58	0.72	0.48	1.09
			24	64	86	0.65*	0.47	0.92
			36	77	102	0.63*	0.46	0.86
			48	84	110	0.61*	0.46	0.82
Pancreatic cancer	29,373	113	12	25	28	0.83	0.46	1.48
			24	35	37	0.82	0.50	1.35
			36	41	42	0.80	0.51	1.27
			48	43	45	0.75	0.48	1.16
Lung cancer	34,489	174	12	22	72	0.29*	0.17	0.47
			24	36	83	0.39*	0.26	0.59
			36	42	92	0.40*	0.27	0.58
			48	45	96	0.39*	0.27	0.57
Melanoma	2,079	0	12	0	0	—	—	—
			24	0	0	—	—	—
			36	0	0	—	—	—
			48	0	0	—	—	—
Breast cancer	20,740	26	12	7	6	1.12	0.32	4.03
			24	7	8	0.81	0.25	2.56
			36	9	10	0.80	0.29	2.20
			48	9	13	0.59	0.22	1.50
Uterine cancer	47,573	36	12	7	11	0.62	0.20	1.75
			24	13	12	1.03	0.43	2.47
			36	14	14	0.93	0.41	2.10
			48	16	17	0.85	0.40	1.79
Ovarian cancer	19,678	20	12	3	5	0.57	0.09	2.95
			24	4	6	0.61	0.13	2.59
			36	4	7	0.51	0.11	2.00
			48	6	8	0.64	0.18	2.11
Prostate cancer	28,039	116	12	22	45	0.46*	0.27	0.79
			24	29	53	0.50*	0.30	0.79
			36	32	64	0.43*	0.27	0.67
			48	34	65	0.44*	0.28	0.67
Bladder cancer	20,835	58	12	18	9	1.81	0.77	4.57
			24	27	14	1.61	0.81	3.32
			36	34	16	1.66	0.89	3.21
			48	36	16	1.66	0.90	3.21
Hematological malignancies	14,177	45	12	13	15	0.80	0.35	1.79
			24	13	19	0.58	0.27	1.25
			36	15	24	0.50	0.24	1.00
			48	16	25	0.49*	0.24	0.95

ASR, adjusted sequence ratio; CI, confidence interval. *: significant inverse signal, —: no detected.

All patients who initiated new treatment with digoxin and whose first diagnosis of cancer was within 48-months period were identified.

Incident users: Number of patients who received their first prescription for digoxin.

Cases with cancer: Number of patients newly diagnosed with cancer.

Diagnosis of cancer last: Number of patients with a diagnosis made after digoxin use.

Diagnosis of cancer first: Number of patients with a diagnosis made before digoxin use.

### Pathway enrichment analysis revealed biogroups associated with canonical pathways inversely correlated between digoxin and cancers

**Figure 1:**
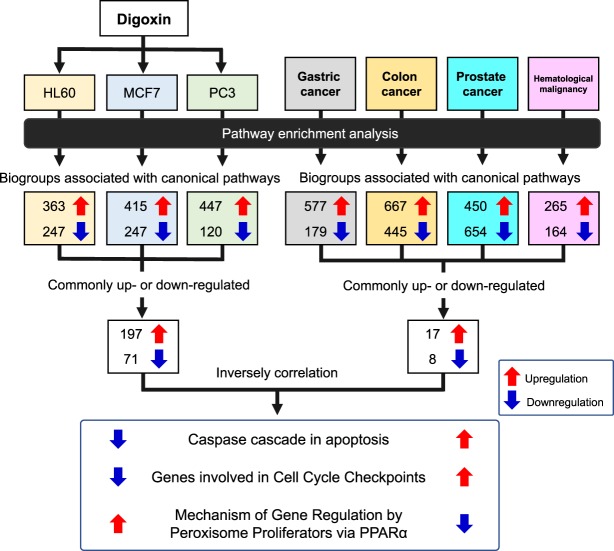
Pathway enrichment analysis using the BaseSpace Correlation Engine database. Human cancer cell lines: HL60, MCF7 and PC3. PPARα, peroxisome proliferator-activated receptor α.

Substantial similarities and some differences in biogroups associated with canonical pathways were identified when differentially expressed genes (DEGs) from three cancer cell lines (HL60, MCF7 and PC3) treated with digoxin or four cancers (gastric cancer, colon cancer, prostate cancer and haematological malignancy) compared with normal tissue were analysed using the BaseSpace Correlation Engine [BSCE, Illumina Inc., CA, USA] (Fig. 1). Pathway enrichment analysis showed that 197 upregulated and 71 downregulated pathways among the three cancer cell lines were commonly observed after digoxin treatment. On the other hand, 17 upregulated and 8 downregulated pathways were commonly observed among the four cancers. Of the inversely correlated canonical pathways between digoxin and cancers, the “Caspase cascade in apoptosis,” “Genes involved in Cell Cycle Checkpoints,” and “Mechanism of Gene Regulation by Peroxisome Proliferators via PPARα” were observed.

### Computational identification of drugs/compounds associated with digoxin

**Figure 2:**
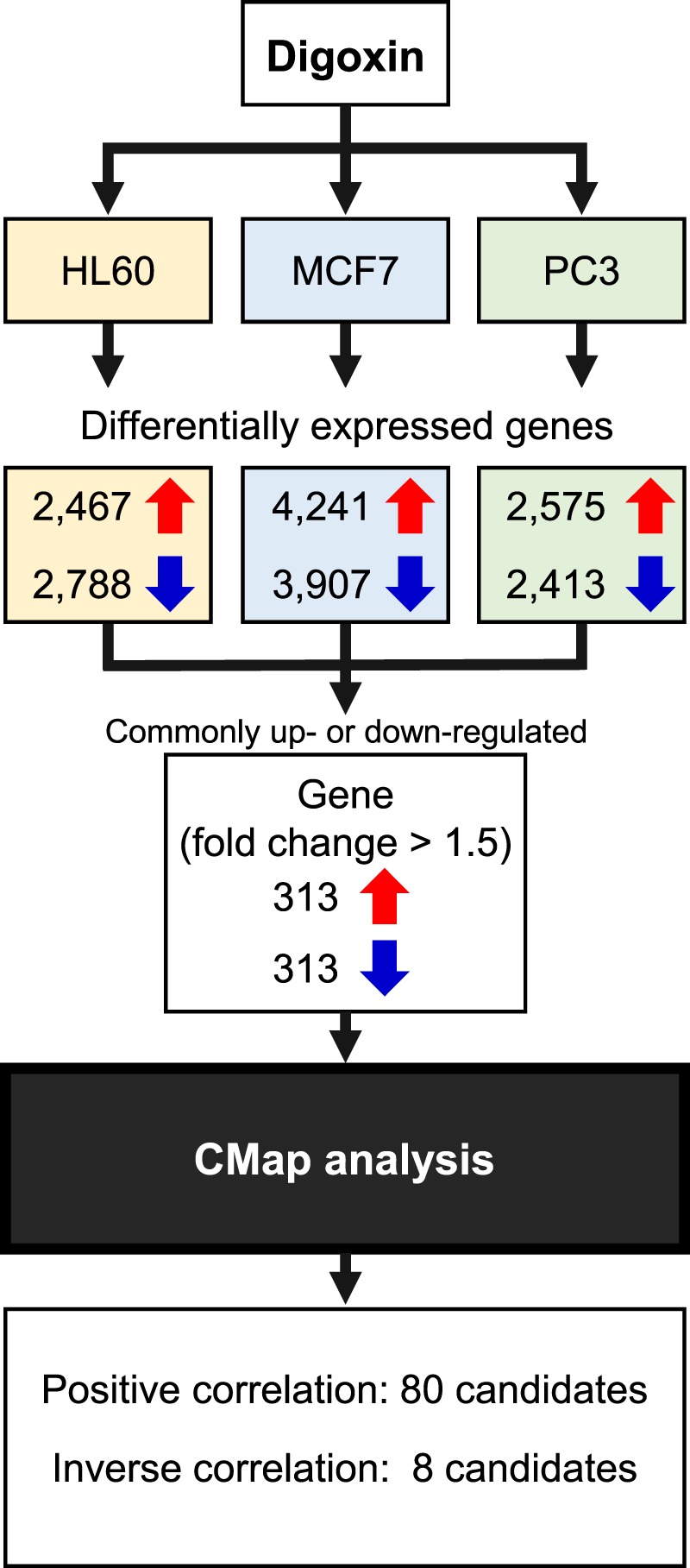
Computational identification of drugs/compounds associated with digoxin using Connectivity Map (CMap). Human cancer cell lines: HL60, MCF7 and PC3. Red arrows, upregulated; blue arrows, downregulated.

After digoxin treatment of the three cancer cell lines (HL60, MCF7 and PC3), 313 upregulated and 313 downregulated genes were found in common (Fig. 2). Connectivity Map analysis of DEGs showed that 80 drugs/compounds were found to be positively correlated (connectivity score: >0) with gene expression signatures for digoxin and eight were inversely correlated (connectivity score: <0; Table 3). In high connectivity score compounds/drugs, cardiac glycosides, such as digoxin, proscillaridin and lanatoside C, were detected. In addition, several anticancer agents (etoposide, vorinostat, carmustine and lomustine) were identified in the 80 drugs/compounds.

**Table 3:** CMap analysis for compounds with gene expression signature for digoxin.

No.	CMap name	Connectivity score
		(mean)
1	digoxin	0.961
2	proscillaridin	0.958
3	lanatoside C	0.949
4	ouabain	0.939
5	digitoxigenin	0.926
6	helveticoside	0.914
7	digoxigenin	0.888
8	strophanthidin	0.703
9	bisacodyl	0.599
10	anisomycin	0.582
11	MG-262	0.531
12	terfenadine	0.497
13	calmidazolium	0.474
14	menadione	0.472
15	niclosamide	0.453
16	cicloheximide	0.448
17	Piperlongumine	0.446
18	1,4-chrysenequinone	0.444
19	pyrvinium	0.433
20	suloctidil	0.431
21	mefloquine	0.429
22	prenylamine	0.428
23	astemizole	0.427
24	parthenolide	0.421
25	fendiline	0.408
26	thioridazine	0.404
27	spiperone	0.388
28	securinine	0.387
29	5224221	0.379
30	phenoxybenzamine	0.375
31	puromycin	0.359
32	STOCK1N-35696	0.350
33	disulfiram	0.328
34	STOCK1N-35874	0.327
35	perhexiline	0.324
36	15-delta prostaglandin J2	0.322
37	metergoline	0.322
38	bepridil	0.319
39	withaferin A	0.312
40	tonzonium bromide	0.308
41	scriptaid	0.306
42	metixene	0.304
43	5182598	0.300
44	pimozide	0.295
45	lycorine	0.292
46	loperamide	0.291
47	beta-escin	0.289
48	alexidine	0.283
49	thiostrepton	0.279
50	AG-028671	0.272
51	proadifen	0.265
52	etoposide	0.258
53	cromoglicic acid	0.257
54	methylbenzethonium chloride	0.256
55	trifluoperazine	0.252
56	hydroquinine	0.244
57	pizotifen	0.243
58	nocodazole	0.239
59	hycanthone	0.238
60	prochlorperazine	0.238
61	dicycloverine	0.231
62	geldanamycin	0.228
63	azacyclonol	0.227
64	desipramine	0.217
65	perphenazine	0.217
66	0179445-0000	0.215
67	antazoline	0.213
68	5155877	0.205
69	alvespimycin	0.204
70	emetine	0.200
71	LY-294002	0.199
72	vorinostat	0.188
73	carmustine	0.186
74	lomustine	0.178
75	gossypol	0.168
76	dihydroergocristine	0.161
77	maprotiline	0.161
78	pergolide	0.149
79	fluphenazine	0.140
80	benzamil	0.111
81	pronetalol	−0.249
82	esculin	−0.267
83	ketorolac	−0.275
84	dydrogesterone	−0.322
85	epitiostanol	−0.333
86	caffeic acid	−0.337
87	buflomedil	−0.342
88	kaempferol	−0.545

CMap: connectivity map.

**Figure 3:**
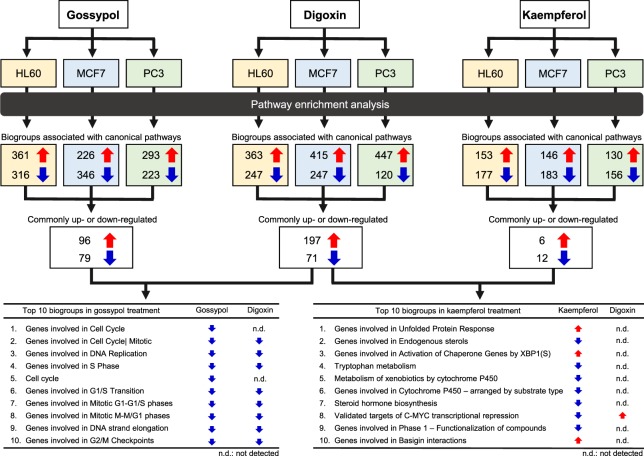
Validation of biogroups associated with canonical pathways of digoxin for anticancer properties. Human cancer cell lines: HL60, MCF7 and PC3. Red arrows, upregulated; blue arrows, downregulated.

According to the results of pathway enrichment analysis with digoxin and the four cancers (gastric cancer, colon cancer, prostate cancer and haematological malignancy), we searched for compounds that influenced the “caspase cascade” and “cell cycle checkpoints” in the 80 drugs/compounds and focused on gossypol, which has been reported to induce apoptosis *via* the caspase cascade^24^. Pathway enrichment analyses in the BSCE database using gossypol identified biogroups associated with canonical pathways that were commonly regulated in the three cancer cell lines. Biogroups ranked in the top 10 in order of high score are shown in Fig. 3. In the same way, biogroups associated with digoxin were identified. By comparing the biogroups derived from digoxin analysis with the top 10 for gossypol, eight out of 10 biogroups were matched. As a negative control, we selected kaempferol, which had the lowest connectivity score of the eight matched drugs/compounds. The results of pathway enrichment analysis revealed the top 10 biogroups associated with canonical pathways of kaempferol were not matched with those of digoxin.

## Discussion

In our analyses of both the FAERS and JMDC claims databases, significant inverse associations were found between digoxin and four cancers (gastric, colorectal, prostate cancer and haematological malignancy). Consistent findings from independent analyses involving different databases, methodologies and algorithms suggest that digoxin use is inversely associated with the risks of these cancers. Of note, there were no significant positive associations between digoxin and any of the investigated cancers. Analysis of the BSCE database suggested the molecular mechanisms by which digoxin suppresses the proliferation of cancer cells include the caspase cascade in apoptosis and peroxisome proliferator-activated receptor α (PPARα) pathways. Thus, these data mining analyses suggest that digoxin may have potential anticancer effects against several cancers.

If digoxin had an influence on cancers in a clinical setting, signals could have been observed by analysis of large-scale clinical data. Analyses of the FAERS and JMDC claims databases revealed significant inverse signals between digoxin and the four cancers. Previous basic research studies have supported the potential value of cardiac glycosides, such as digoxin, as chemotherapeutic candidates^25–28^. At the mechanistic level, several different pathways have been suggested to be responsible for mediating cytotoxic effects, including calcium-dependent activation of caspases and other hydrolytic enzymes^5,29^, generation of reactive oxygen species^30^, topoisomerase inhibition^31^ and interference of signal transduction pathways, such as Src, epidermal growth factor receptor, p21 and hypoxia inducible factor-1α^2,4^. In the present study, data mining was used to elucidate the molecular mechanisms of digoxin. In reference to the signature reversion strategy^32^, biogroups associated with canonical pathways can be screened on the basis of the chance overlap between the gene expression signature of digoxin and those of cancers. We detected inversely regulated biogroups associated with canonical pathways between digoxin and the four cancers. The matching between digoxin and cancer profiles revealed three canonical pathways, which included the “Caspase cascade in apoptosis.” Previous reports have shown that digoxin has apoptosis-related function^5,29^. Furthermore, the associated PPARα pathway was also noted. PPARα is known to be involved in fatty acid metabolism and fatty acid transport and metabolism are associated with metastatic progression and poor prognosis of cancers^33^. Several studies using cancer cell lines have shown PPARα as a biomarker^34,35^. Thus, digoxin may have a novel ability to suppress cancer invasiveness and progression *via* pathways associated with PPARα.

In the present study, no significant associations were observed between digoxin and several cancers such as breast or uterine cancers. When three, significant, inverse signals (ROR, IC and adjusted SR) were observed in our analysis, we considered that the criteria of significant inverse association were satisfied. Therefore, the reliability of inverse association derived from integrative analysis using DPA and SSA seemed to be high. Digoxin has been reported to be a risk factor of breast cancer as it binds to the oestrogen receptor^8,36^. Breast and endometrial cancers are oestrogen-dependent cancers. Therefore, the oncogenic property rather than the anticancer property of digoxin is concerning. As a result, our analysis did not detect significant inverse association between digoxin and oestrogen-dependent cancers. These findings support the usefulness of our integrative approach.

If digoxin has antiproliferative activity, the identified digoxin-induced DEGs should match with those induced by anticancer agents. The result of Connectivity Map analysis suggests several anticancer agents (etoposides, vorinostat, carmustine and lomustine) that are generally used in clinical settings. These drugs have been known to cause cell cycle arrest and to induce apoptosis. Gossypol also has DEG signatures similar to that of digoxin. Eight out of the top 10 biogroups associated with the canonical pathways of gossypol, which were matched to that of digoxin, were mainly concerned with the cell cycle. Thus, digoxin may have the ability to cause cell cycle arrest followed by apoptosis.

There are several inherent limitations associated with FAERS analysis such as reporting bias or unmeasured confounders^37^. Under-reporting or selective reporting are included. Certainly, not all adverse events observed in clinical settings are included in the database. As FAERS database contains missing data, misspelled drug names or duplicate data, we had deleted or corrected such data before conducting analysis in this study. Several variables were also limited in our FAERS analysis. Age, sex, race, treatment duration, drug dosage or co-administered drugs were not considered. It is generally recognised that oncogenesis occurs over a long period. Thus, the duration of digoxin treatment may be an important factor for suppressing oncogenesis. However, the duration of digoxin treatment was not available in the FAERS database. Although these aforementioned factors may affect the results of DPA, DPA using spontaneous reporting systems is useful for detecting the potential signals of drugs. We confirmed the robustness of the signals by analysing the common signals using two different algorithms: ROR and IC. However, signal detection using DPA should not be interpreted as assuming a causal relation between drugs and clinical events. Basically, DPA cannot be used for estimating the causative factors or the comparative risk of any drugs owing to the lack of denominators in the database. The hypotheses generated by DPA needs to be validated using other methods. In the next step, a different method, event SSA (ESSA), and a different database, JMDC claims database, were used in our analysis. ESSA has been used to detect the association between drug exposure and the outcome of interest as it has demonstrated moderate sensitivity and high specificity for signal detection^38^. However, this method also has potential limitations such as varying prescription trends, time-varying confounders, detection bias or confounding by indication^39^. In the present study, we used JMDC claims database for conducting ESSA. In this database, the proportion of elderly patients aged ≥65 years is low as this database has been built by the employees’ health insurance system. The prevalence of cancer generally increases with increasing age; therefore, this restriction in JMDC claims database may reduce the generalizability of results. The diagnoses contained in the claims database were not validated, and cancer diagnosis was broadly classified in our analysis. There exist phenotypic and functional heterogeneities in cancers. For example, lung cancer is divided into non-small cell lung cancer and small cell lung cancer and uterine cancer into cervical and endometrial cancers. Furthermore, breast cancer has been classified into some subtypes. Thus, more detailed analyses are necessary for each cancer type. Detailed classification of cancers leads to reduce sample size. Therefore, a big database will be needed. In our analysis, the maximum interval duration of ESSA was 48 months. As oncogenesis requires long duration, it may be better to prolong an interval more. Reportedly, during the 10-year follow-up period, an increased risk of cancer was observed in patients receiving digoxin^40^. Another report demonstrated that the risk of cancers associated with both long- and short-term digoxin uses was controversial^41^. Using longer intervals for ESSA would introduce other confounding factors and lead to false positive signals^42^. A careful interpretation of the results is required if the interval is extended. Herein, the BSCE database was utilised to estimate new molecular mechanisms of digoxin in cancers. We speculated molecular mechanisms using the transcriptome database composed of *in-vitro* studies; however, these mechanisms may differ from mechanisms occurring in human body. Indeed, no scientific approach is all-powerful; the same is true of *in silico* approaches. As a basic premise to incorporate *in silico* approaches in drug evaluation, it should be emphasised that they are not a substitute for *in vivo* experiments and should be performed in parallel to basic or clinical studies.

The results of our integrative analysis using different methodologies, algorithms and large-scale databases suggests that digoxin use is inversely associated with at least four cancers. Furthermore, the possibility of digoxin’s anticancer effect *via* PPARα and apoptotic caspase cascade pathways was suggested. Although several studies have suggested that digoxin is a potential candidate anticancer agent^43–45^, no definitive evidence exists yet. Our results provide a framework for uncovering and validating previously overlooked/undetected associations between digoxin use and anticancer effects by using both clinical and bioinformatics databases. Further basic research and epidemiological studies are required to confirm our findings.

## Materials and Methods

### Analysis of the FAERS database

The FAERS clinical database was accessed from the US Food and Drug Administration’s website (http://www.fda.gov/Drugs/GuidanceComplianceRegulatoryInformation/Surveillance/AdverseDrugEffects/). The present study included data from the first quarter of 2004 through the end of 2016. A total of 99,108,600 reports were obtained. Preferred terms from the Medical Dictionary for Regulatory Activities (MedDRA^®^ version 20.1) were used to classify adverse events.

Arbitrary drug names, including trade names and abbreviations, were mapped into unified generic names *via* text mining using the Martindale website (https://www.medicinescomplete.com/mc/login.htm). Digoxin was identified by linking this archive with the FAERS database. All records that included digoxin in DRUG files were selected, and relevant reactions from REACTION files were then identified. Adverse events in the FAERS database were coded using MedDRA^®^ preferred terms, which are grouped by defined medical conditions of the area of interest. We identified preferred terms related to cancers using Standardized MedDRA^®^ queries. Preferred terms were linked to High-Level Terms (Table S2).

ROR and IC in DPA were utilised to detect spontaneous report signals. ROR and IC with a 95% confidence interval (CI) were calculated according to methods previously described^17^. Briefly, signal scores were calculated using a case/non-case method. Those reports containing the event of interest were defined as cases; all other reports comprised the non-cases. For the ROR, an inverse signal was defined if the upper limit of the 95% two-sided CI was less than 1. For the IC, an inverse signal was defined if the upper limit of the 95% CI was less than 0. In the current study, two methods were used to detect inverse signals, and the association between digoxin use and the diagnosis of cancers was listed as an inverse signal (drug repositioning signal) when the two indices met the criteria outlined above.

### Analysis of the JMDC claims database

The JMDC claims database is a large and chronologically organised claims database constructed by the JMDC Co., Ltd.^46^ using standardised disease classifications and anonymous record linkage. In total, this database includes approximately 3.8 million insured persons in Japan (approximately 3.2% of the population) and mainly comprises company employees and their family members. This database provides information on the beneficiaries, including encrypted personal identifiers, age, sex, International Classification of Diseases, 10^th^ revision, procedure and diagnostic codes, as well as the name, dose and number of days supplied of the prescribed and/or dispensed drugs. All drugs were coded according to the Anatomical Therapeutic Chemical Classification of the European Pharmaceutical Market Research Association. An encrypted personal identifier was used to link claims data from different hospitals, clinics and pharmacies.

ESSA was performed to evaluate the association between digoxin use and cancers, and adjusted sequence ratios (SRs) were calculated with reference to a previous report^18^. Briefly, the ESSA evaluates asymmetry in the distribution of an incident event before and after initiation of a specific treatment. Asymmetry may indicate an association between a specific treatment of interest and the event^42,47^. The crude SR is defined as the ratio of the number of newly diagnosed cancer patients after initiation of digoxin relative to the number before initiation. In addition, the SRs were adjusted for temporal trends in digoxin and events using the method proposed by Hallas^47^. The probability that digoxin was prescribed first, in the absence of any causal relationship, can be estimated by a so-called null-effect SR. The null-effect SR generated by the proposed model may be interpreted as a reference value for the SR. Therefore, the null-effect SR is the expected SR in the absence of any causal association after accounting for incidence trends. By dividing the crude SR by the null-effect SR, an adjusted SR corrected for temporal trends is obtained. A slightly modified model was used to account for the limited time interval allowed between digoxin use and cancer diagnosis. In the present study, an adjusted SR of less than 1 was defined as an inverse association between digoxin use and risk of cancer (drug repositioning signal).

All incident users of digoxin and all newly diagnosed cancer cases were identified from January 2005 to February 2015. Target cancers were defined according to International Classification of Disease, 10^th^ revision, codes (Table S3). Those patients were followed up until February 2015. Incidence was defined as the first prescription of digoxin. To exclude prevalent users of digoxin, the analysis was restricted to users whose first prescription was administered in July 2005 or later (after a run-in period of 6 months). Likewise, the analysis was restricted to cases whose first diagnosis was in July 2005 or later. Waiting time distribution analysis was performed to ensure that the analysis was restricted to incident users of digoxin and newly diagnosed cases of cancer^48^. An identical run-in period was also applied to patients enrolled in the cohort after June 2005. Incident users were identified by excluding those patients who received their first digoxin prescription before July 2005, and newly diagnosed cancer cases were identified by excluding those patients whose first diagnosis of cancer was before July 2005. Those patients who had initiated a new treatment with digoxin and whose first diagnosis of cancer was within 12, 24, 36 and 48 month periods (intervals) of treatment initiation were identified. Patients who had received their first digoxin prescription and whose first cancer diagnosis was within the same month were not included in determination of the SR. The 95% CI for the adjusted SR was calculated using a method for exact CIs for binomial distributions^49^.

### Pathway enrichment analysis using the BSCE database

The BSCE bioinformatics database was used to investigate microarray gene expression profiles. This engine can search curated gene expression profiles of compounds or diseases of interest available from an open source: Gene Expression Omnibus. Target drugs/compounds and cancer names were used as queries to filter gene expression datasets of subsequent gene expression profiling. For each query term, we identified microarray datasets that met the inclusion criteria of differential mRNA expression data for humans acquired by analyses of a perturbed condition and unaffected control with a high signal-to-noise ratio. We utilised publicly available microarray datasets shown in Table S4. Gene expression datasets (i.e. biosets) were extracted from the BSCE database and subjected to pathway enrichment analysis. To profile the results of pathway enrichment analysis, we performed text mining and used curated pathways. We derived commonly differential regulated pathways when the query drug/compound was administered with three human-derived cancer cell lines (HL60, MCF7 and PC3). We also derived differentially regulated pathways in four cancers (gastric cancer, colorectal cancer, prostate cancer and haematological malignancy) compared with control. If a drug/compound had a signature with up- or downregulated pathways opposite that of a disease signature (down- or upregulated pathways), that drug/compound had a molecular mechanism involving these pathways and could potentially be used as a treatment for that disease.

### Combination analysis using the BSCE database and Connectivity Map

We focused on DEGs induced by target drug/compound administration in HL60, MCF7 and PC3 cell lines. DEG probes commonly expressed in these three cell lines with a *P* < 0.05 and |Fold change| > 1.5 were obtained from the BSCE database, and these probes were included in the Connectivity Map analysis^50^. When the reference signature for a drug matched the digoxin signature with a *P* < 0.05, it was short-listed with the connectivity score to indicate its correlation with a query drug/compound.

### Data management

Data management and analysis were performed using Visual Mining Studio software (version 8.1; NTT DATA Mathematical Systems Inc., Tokyo, Japan). Quantitative data are expressed as means ± standard deviations and categorical data as frequencies (percentages).

## Supplementary information

Supplementary information

## Data Availability

All data generated or analysed during this study are included in this published article and its supplementary information files.

## References

[1] Orrenius S, Zhivotovsky B, Nicotera P (2003). Regulation of cell death: the calcium-apoptosis link. Nat. Rev. Mol. Cell Biol..

[2] Kometiani P, Liu L, Askari A (2005). Digitalis-induced signaling by Na+/K+-ATPase in human breast cancer cells. Mol. Pharmacol..

[3] Lin H, Juang JL, Wang PS (2004). Involvement of Cdk5/p25 in digoxin-triggered prostate cancer cell apoptosis. J. Biol. Chem..

[4] Zhang H (2008). Digoxin and other cardiac glycosides inhibit HIF-1alpha synthesis and block tumor growth. Proc. Natl. Acad. Sci. USA.

[5] Winnicka K, Bielawski K, Bielawska A (2006). Cardiac glycosides in cancer research and cancer therapy. Acta Pol. Pharm..

[6] Biggar RJ (2012). Molecular pathways: digoxin use and estrogen-sensitive cancers–risks and possible therapeutic implications. Clin. Cancer Res..

[7] Biggar RJ, Wohlfahrt J, Melbye M (2012). Digoxin use and the risk of cancers of the corpus uteri, ovary and cervix. Int. J. Cancer.

[8] Biggar RJ, Wohlfahrt J, Oudin A, Hjuler T, Melbye M (2011). Digoxin use and the risk of breast cancer in women. J. Clin. Oncol..

[9] Ahern TP, Lash TL, Sorensen HT, Pedersen L (2008). Digoxin treatment is associated with an increased incidence of breast cancer: a population-based case-control study. Breast Cancer Res..

[10] Biggar RJ, Andersen EW, Kroman N, Wohlfahrt J, Melbye M (2013). Breast cancer in women using digoxin: tumor characteristics and relapse risk. Breast Cancer Res..

[11] Ashburn TT, Thor KB (2004). Drug repositioning: identifying and developing new uses for existing drugs. Nat Rev Drug Discov.

[12] Qu XA, Rajpal DK (2012). Applications of Connectivity Map in drug discovery and development. Drug Discov Today.

[13] Kupershmidt I (2010). Ontology-based meta-analysis of global collections of high-throughput public data. PLoS One.

[14] Lipinski C, Hopkins A (2004). Navigating chemical space for biology and medicine. Nature.

[15] Lussier YA, Chen JL (2011). The emergence of genome-based drug repositioning. Sci. Transl. Med..

[16] Wahab, I. A., Pratt, N. L., Kalisch, L. M. & Roughead, E. E. Sequence Symmetry Analysis and Disproportionality Analyses: What Percentage of Adverse Drug Reaction do they Signal? *Advances in Pharmacoepidemiology & Drug Safety*, **02** (2013). 10.1002/pds.3417 23412832

[17] Takada M, Fujimoto M, Motomura H, Hosomi K (2016). Inverse Association between Sodium Channel-Blocking Antiepileptic Drug Use and Cancer: Data Mining of Spontaneous Reporting and Claims Databases. Int. J. Med. Sci..

[18] Nagashima T, Shirakawa H, Nakagawa T, Kaneko S (2016). Prevention of antipsychotic-induced hyperglycaemia by vitamin D: a data mining prediction followed by experimental exploration of the molecular mechanism. Sci. Rep..

[19] Horinouchi Y (2018). Renoprotective effects of a factor Xa inhibitor: fusion of basic research and a database analysis. Sci. Rep..

[20] Wang K, Wan M, Wang RS, Weng Z (2016). Opportunities for Web-based Drug Repositioning: Searching for Potential Antihypertensive Agents with Hypotension Adverse Events. J. Med. Internet Res..

[21] Chen YA (2018). Simvastatin Therapy for Drug Repositioning to Reduce the Risk of Prostate Cancer Mortality in Patients With Hyperlipidemia. Front. Pharmacol..

[22] Vargas DM, De Bastiani MA, Zimmer ER, Klamt F (2018). Alzheimer’s disease master regulators analysis: search for potential molecular targets and drug repositioning candidates. Alzheimers Res. Ther..

[23] Hosomi K (2018). An integrative approach using real-world data to identify alternative therapeutic uses of existing drugs. PLoS One.

[24] Zhang XQ (2010). Inhibition of proliferation of prostate cancer cell line, PC-3, *in vitro* and *in vivo* using (-)-gossypol. Asian J Androl.

[25] Hu Y (2018). Lanatoside C inhibits cell proliferation and induces apoptosis through attenuating Wnt/beta-catenin/c-Myc signaling pathway in human gastric cancer cell. Biochem. Pharmacol..

[26] Felth J (2009). Cytotoxic effects of cardiac glycosides in colon cancer cells, alone and in combination with standard chemotherapeutic drugs. J. Nat. Prod..

[27] Juang HH, Lin YF, Chang PL, Tsui KH (2010). Cardiac glycosides decrease prostate specific antigen expression by down-regulation of prostate derived Ets factor. J. Urol..

[28] Zhang XH (2017). The combination of digoxin and GSK2606414 exerts synergistic anticancer activity against leukemia *in vitro* and *in vivo*. Biofactors.

[29] Schoner W, Scheiner-Bobis G (2007). Endogenous and exogenous cardiac glycosides: their roles in hypertension, salt metabolism, and cell growth. Am. J. Physiol. Cell Physiol..

[30] Newman RA (2006). Oleandrin-mediated oxidative stress in human melanoma cells. J. Exp. Ther. Oncol..

[31] Bielawski K, Winnicka K, Bielawska A (2006). Inhibition of DNA topoisomerases I and II, and growth inhibition of breast cancer MCF-7 cells by ouabain, digoxin and proscillaridin A. Biol. Pharm. Bull..

[32] Iorio F, Rittman T, Ge H, Menden M, Saez-Rodriguez J (2013). Transcriptional data: a new gateway to drug repositioning?. Drug Discov Today.

[33] Nath A, Chan C (2016). Genetic alterations in fatty acid transport and metabolism genes are associated with metastatic progression and poor prognosis of human cancers. Sci. Rep..

[34] Zhao Z (2014). Nestin positively regulates the Wnt/beta-catenin pathway and the proliferation, survival and invasiveness of breast cancer stem cells. Breast Cancer Res..

[35] Drakaki A (2015). Functional microRNA high throughput screening reveals miR-9 as a central regulator of liver oncogenesis by affecting the PPARA-CDH1 pathway. BMC Cancer.

[36] Rifka SM, Pita JC, Loriaux DL (1976). Mechanism of interaction of digitalis with estradiol binding sites in rat uteri. Endocrinology.

[37] Sakaeda T, Tamon A, Kadoyama K, Okuno Y (2013). Data mining of the public version of the FDA Adverse Event Reporting System. Int. J. Med. Sci..

[38] Wahab IA, Pratt NL, Wiese MD, Kalisch LM, Roughead EE (2013). The validity of sequence symmetry analysis (SSA) for adverse drug reaction signal detection. Pharmacoepidemiol. Drug Saf..

[39] Lai EC-C (2017). Sequence symmetry analysis in pharmacovigilance and pharmacoepidemiologic studies. Eur. J. Epidemiol..

[40] Chung MH, Wang YW, Chang YL, Huang SM, Lin WS (2017). Risk of cancer in patients with heart failure who use digoxin: a 10-year follow-up study and cell-based verification. Oncotarget.

[41] Xie SH, Jernberg T, Mattsson F, Lagergren J (2017). Digitalis use and risk of gastrointestinal cancers: A nationwide population-based cohort study. Oncotarget.

[42] Tsiropoulos I, Andersen M, Hallas J (2009). Adverse events with use of antiepileptic drugs: a prescription and event symmetry analysis. Pharmacoepidemiol. Drug Saf..

[43] Fujii T (1864). Crosstalk between Na(+),K(+)-ATPase and a volume-regulated anion channel in membrane microdomains of human cancer cells. Biochim Biophys Acta Mol Basis Dis.

[44] Wang T (2017). Effects of digoxin on cell cycle, apoptosis and NF-kappaB pathway in Burkitt’s lymphoma cells and animal model. Leuk. Lymphoma.

[45] Lin SY (2015). Digoxin Suppresses Tumor Malignancy through Inhibiting Multiple Src-Related Signaling Pathways in Non-Small Cell Lung Cancer. PLoS One.

[46] Kimura S, Sato T, Ikeda S, Noda M, Nakayama T (2010). Development of a database of health insurance claims: standardization of disease classifications and anonymous record linkage. J. Epidemiol..

[47] Hallas J (1996). Evidence of depression provoked by cardiovascular medication: a prescription sequence symmetry analysis. Epidemiology.

[48] Hallas J, Gaist D, Bjerrum L (1997). The waiting time distribution as a graphical approach to epidemiologic measures of drug utilization. Epidemiology.

[49] Morris JA, Gardner MJ (1988). Calculating confidence intervals for relative risks (odds ratios) and standardised ratios and rates. Br. Med. J. (Clin. Res. Ed).

[50] Lamb J (2006). The Connectivity Map: using gene-expression signatures to connect small molecules, genes, and disease. Science.

