# Supplementary material for: Integrative analysis of clinical and bioinformatics databases to identify anticancer properties of digoxin
Source: Sci Rep. 2019 Nov 12;9:16597. doi: 10.1038/s41598-019-53392-y (PMC6851125; doi:10.1038/s41598-019-53392-y)
Supplement: Supplementary file 1 — Supplementary information [file 41598_2019_53392_MOESM1_ESM.pdf]

Integrative analysis of clinical and bioinformatics databases to identify anticancer properties of digoxin

Satoshi Yokoyama*, Yasuhiro Sugimoto, Chihiro Nakagawa, Kouichi Hosomi, Mitsutaka Takada

Division of Clinical Drug Informatics, School of Pharmacy, Kindai University, 3-4-1 Kowakae, Higashiosaka City, Osaka 577-8502, Japan

Correspondence: Satoshi Yokoyama, Ph. D., Division of Clinical Drug Informatics, School of Pharmacy, Kindai University, 3-4-1 Kowakae, Higashi-osaka, Osaka, 577-8502, Japan.
Telephone number: +81-6-6721-2332
Fax number: +81-6-6730-1394
E-mail address: *yokoyama@phar.kindai.ac.jp

Supplementary Information

Table S1. Characteristics of this study population for digoxin users from January 2005 to Feburuary 2015

Table S2. Definitions of cancers according to HLT in MedDRA

Table S3. Defined cancers according to ICD-10 code

Table S4. Microarray datasets used in this study

Table S1. Characteristics of this study population for digoxin users from January 2005 to Feburuary 2015

	Total	Male	Female
Digoxin users, n	3,035		
Claims including digoxin, n	52,828		
Incident users, n (%)	1,297	878 (67.7)	419 (32.3)
Age, years, n (%)			
<20	90 (6.9)	48 (5.5)	42 (10.0)
20-39	137 (10.6)	88 (10.0)	49 (11.7)
40-59	544 (41.9)	397 (45.2)	147 (35.1)
60-79	526 (40.6)	345 (39.3)	181 (43.2)
≧80	0 (0.0)	0 (0.0)	0 (0.0)
Mean ±S.D.	52.0±17.4	52.4±15.9	51.3±20.1

Incident users: number of patients who received their first prescription for digoxin

S.D.: standard deviation

Table S2. Definitions of cancers according to HLT in MedDRA

Cancers	HLT
Esophageal cancer	Oesophageal neoplasms malignant
Gastric cancer	Gastric neoplasms malignant
Colorectal cancer	Colorectal neoplasms malignant
Pancreatic cancer	Pancreatic neoplasms malignant (excl islet cell and carcinoid)
Lung cancer	Non-small cell neoplasms malignant of the respiratory tract cell type specified<br>Respiratory tract small cell carcinomas
Melanoma	Skin melanomas (excl ocular)
Breast cancer	Breast and nipple neoplasms malignant
Uterine cancer	Cervix neoplasms malignant<br>Endometrial neoplasms malignant<br>Uterine neoplasms malignant NEC
Ovarian cancer	Ovarian germ cell neoplasms malignant<br>Ovarian neoplasms malignant (excl germ cell)
Prostate cancer	Prostatic neoplasms malignant
Bladder cancer	Bladder neoplasms malignant
Hematological malignancies	Adult T-cell lymphomas/leukaemias<br>Anaplastic large cell lymphomas T- and null-cell types<br>Angiocentric lymphomas<br>Angioimmunoblastic T-cell lymphomas<br>B-cell lymphomas NEC<br>B-cell small lymphocytic lymphomas<br>B-cell unclassifiable lymphomas<br>Burkitt's lymphomas<br>Diffuse large B-cell lymphomas<br>Extranodal marginal zone B-cell lymphomas (low grade B-cell)<br>Follicle centre lymphomas diffuse predominantly small cell<br>Follicle centre lymphomas| follicular grade I| II| III<br>High grade B-cell lymphomas Burkitt-like lymphoma<br>Hodgkin's disease NEC<br>Hodgkin's disease lymphocyte depletion type<br>Hodgkin's disease lymphocyte predominance type<br>Hodgkin's disease mixed cellularity type<br>Hodgkin's disease nodular sclerosis type<br>Intestinal T-cell lymphomas<br>Leukaemias NEC<br>Leukaemias acute NEC<br>Leukaemias acute lymphocytic<br>Leukaemias acute myeloid<br>Leukaemias chronic NEC<br>Leukaemias chronic T-cell<br>Leukaemias chronic lymphocytic<br>Leukaemias chronic myeloid<br>Leukaemias lymphocytic NEC<br>Leukaemias myeloid NEC<br>Lymphomas unclassifiable malignant<br>Lymphomas unspecified NEC<br>Lymphoplasmacytoid lymphomas/immunocytomas<br>Mantle cell lymphomas<br>Marginal zone lymphomas NEC<br>Mycoses fungoides<br>Myelodysplastic syndromes<br>Myeloproliferative disorders (excl leukaemias)<br>Nodal marginal zone B-cell lymphomas<br>Non-Hodgkin's lymphomas NEC<br>Non-Hodgkin's lymphomas transformed recurrent<br>Non-Hodgkin's lymphomas unspecified histology aggressive<br>Non-Hodgkin's lymphomas unspecified histology indolent<br>Peripheral T-cell lymphomas NEC<br>Precursor B-lymphoblastic lymphomas<br>Precursor T-lymphoblastic lymphomas/leukaemias<br>Primary mediastinal large B-cell lymphomas<br>Splenic marginal zone lymphomas<br>T-cell unclassifiable lymphomas<br>Waldenstrom's macroglobulinaemias

MedDRA: Medical Dictionary for Regulatory Activities, HLT: High Level Term.

Table S3. Defined cancers according to ICD-10

Definition	ICD-10 code
Esophageal cancer	C15
Gastric cancer	C16
Colorectal cancer	C18-C20
Pancreatic cancer	C25
Lung cancer	C34
Melanoma	C43
Breast cancer	C50
Uterine cancer	C53-C55
Ovarian cancer	C56
Prostate cancer	C61
Bladder cancer	C67
Hematological malignancies	C81-C85, C88, C90-C96

ICD-10: International classification diseases, tenth revision.

Table S4. Microarray datasets used in this study

Definition	GSE accession (Internal ID)	Sample
Gastric cancer	GSE19826_1	Genes changing in gastric_tumor vs. gastric_tissue_normal_tumor_adjacent.
Colon cancer	GSE10972_1	Genes changing in Colon_cancer_tissue vs. Adjacent_normal_tissue.
Prostate cancer	GSE9347_1	Genes changing in Primary_prostate_cancer vs. Paired_normal_control.
Hematological malignancy	GSE19147_1	Genes changing in CD3+_T-cells_from_peripheral_blood_of_patient_with_B-cell_chronic_lymphocytic_leukemia vs. CD3+_T-

Definition	NextBio Internal ID	Sample
HL60 cells + digoxin	Platform_HT_HG-U133A_Batch_634_1	Genes changing in HL60 cells + digoxin 5.2uM for 6 hrs in DMSO vs. Vehicle alone.
MCF7 cells + digoxin	Platform_HT_HG-U133A_Batch_655_1<br>Platform_HT_HG-U133A_Batch_730_1	Genes changing in MCF7 cells + digoxin 5.2uM for 6 hrs in DMSO vs. Vehicle alone.
PC3 cells + digoxin	Platform_HT_HG-U133A_Batch_715_1	Genes changing in PC3 cells + digoxin 5.2uM for 6 hrs in DMSO vs. Vehicle alone.
HL60 cells + gossypol	Platform_HT_HG-U133A_Batch_645_35	Genes changing in HL60 cells + gossypol 7.8uM for 6 hrs in DMSO vs. Vehicle alone.
MCF7 cells + gossypol	Platform_HT_HG-U133A_Batch_685_31<br>Platform_HT_HG-U133A_Batch_700_33<br>Platform_HT_HG-U133A_Batch_751_36	Genes changing in MCF7 cells + gossypol 7.8uM for 6 hrs in DMSO vs. Vehicle alone.
PC3 cells + gossypol	Platform_HT_HG-U133A_Batch_681_33<br>Platform_HT_HG-U133A_Batch_701_28	Genes changing in PC3 cells + gossypol 7.8uM for 6 hrs in DMSO vs. Vehicle alone.
